# A novel DNA damage-induced alternative splicing pathway that regulates p53 and cellular senescence markers

**DOI:** 10.18632/oncoscience.367

**Published:** 2017-10-12

**Authors:** Jing Chen, Michael B. Kastan

**Affiliations:** Department of Pharmacology and Cancer Biology, Duke University School of Medicine, Durham, NC 27710, USA; Duke Cancer Institute, Duke University, Durham, NC 27710, USA

**Keywords:** DNA damage, alternative splicing, p53, senescence

The integrity of the human genome is constantly threatened by a wide variety of challenges (including radiation, chemicals from the environment, intracellular oxygen radicals, telomere shortening, etc.) [1]. Suboptimal cellular responses to DNA damage and accumulation of damaged cells contribute to cancer development and to aging-associated pathogenesis. Conversely, cellular responses to DNA damage are also important determinants of clinical outcomes in cancer patients since therapeutic radiation and many cancer chemotherapeutics work by damaging DNA. Genotoxic insults activate DNA damage responses (DDR) which induce signal transduction pathways with a myriad of post-translational modifications and alter global gene expression patterns by modulating chromatin structure, transcription and translation programs. These responses affect cell cycle progression, DNA repair, apoptosis, genetic stability, and cellular senescence.

Recent data suggested that the DDR also impacts alternative splicing (AS), a highly regulated process evolved in eukaryotes to diversify their transcriptomes and proteomes [2, 3]. For example, Muñoz et al. found that ultraviolet radiation (UV) slows down global transcription elongation by changing the phosphorylation state of the carboxy-terminal tail of RNA polymerase II and promotes the AS events for proapoptotic genes [4]. However, the mechanisms by which the damage signals are transduced to specific splicing factors to regulate AS and affect cell fate remained to be determined. We recently reported the existence of a novel signaling pathway that is activated by ionizing irradiation (IR) and alters the AS pattern of numerous genes, including the human tumor suppressor gene, p53, and contributes to regulation of damage- induced cellular senescence [5].

We were surprised to discover that IR rapidly inhibits the activity of the SMG1 kinase, an enzyme previously implicated in the regulation of nonsense- mediated RNA decay [6]. This finding was unexpected since other phosphoinositide 3-kinase-related protein kinase (PIKK) family members, including the ATM protein kinase, are activated as part of the DNA damage response [1]. Because of its role in regulating nonsense- mediated RNA decay, SMG1 is often bound to RNA; inactivation of the SMG1 kinase after IR reduces its binding selectively to intronic regions of p53 precursor mRNA near Exon 9. This disruption of SMG1 binding to the intronic regions results in the promotion of the binding of ribosomal protein RPL26 to p53 pre-mRNA and RPL26, in turn, is required for the recruitment of the serine/arginine-rich splicing factor, SRSF7, to p53 pre- mRNA. This DNA damage-induced binding of SRSF7 to p53 pre-mRNA then facilitates the generation of the alternatively spliced p53β mRNA, one of the 12 splice variants of human p53 gene [7].

p53β is generated by retention of a cryptic exon inside intron 9 of the p53 gene via alternative splicing [7]. The retained intron contains a premature stop codon which leads to a replacement of the entire p53 C-terminal oligomerization domain with a short 10 amino acid tail in p53β protein. Like full-length p53, this truncated form of p53 maintains the DNA binding domain and still functions as a transcription factor. As previously reported [8], we found that over-expression of p53β triggers induction of cellular senescence markers. Importantly, selective knockout of p53β by CRISPR/Cas9 significantly reduced IR-induced senescence markers (such as senescence associated β-galactosidase activity, senescence associated secretory phenotype (SASP), etc.), even though the DNA damage still induced full-length p53 and its transcriptional targets, like p21. Down-regulation of molecules involved in the signaling pathway, like SRSF7, also reduced IR- induction of cellular senescence markers. Thus, the pathway “SMG1-RPL26-SRSF7-p53β” seems to transmit a specific signal for cellular senescence after damage.

In exploring the mechanisms by which p53β might be affecting specific cellular outcomes after damage, we found that cells lacking p53β expression fail to transcriptionally repress negative regulators of aging such as BCL6 and SIRT1. Interestingly, both of these two genes epigenetically regulate downstream target gene expression. Therefore, transient induction of p53β after IR may be able to alter cellular phenotypes after damage by regulating epigenetic modifications that drive cells toward a senescence pathway.

Transcriptional profiling revealed over 600 distinct AS events in cells after IR treatment and we speculate that some of these events, like p53β splicing, are regulated by the “SMG1-RPL26-SRSF7” pathway and contribute to senescent phenotypes in damaged cells. In addition to intron retentions (p53β), we also identified some AS events that are generated by alternative exon usages (e.g. the CDYL gene), suggesting a potentially broad range of targets for this pathway. We are in the process of validating some of these events in the hope of identifying novel determinants of cellular aging and potential therapeutic targets for aging-associated diseases and some of the toxic side-effects of irradiation and chemotherapy.

**Figure 1:**
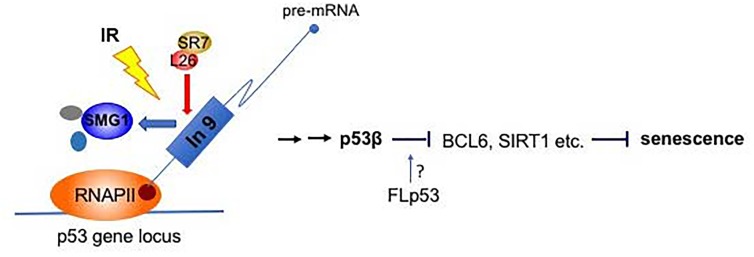
“SMG1-RPL26-SRSF7-p53β” pathway for damage induced cellular senescence IR inhibits SMG1 kinase activity. Inactivated SMG1 reduces its binding to intron 9 region of human p53 pre-mRNA, which allows the recruitment of RPL26 and SRSF7 for p53β splicing. p53β may repress the transcription of anti-aging genes such as BCL-6, SIRT1 etc. and induce cellular senescence. The role played by full-length p53 in this pathway is still undetermined.
